# The Sad End of a Promising Life

**Published:** 1881-02

**Authors:** 


### THE SAD END OF A PROMISING LIFE.

The sad intelligence is just at hand that, on Saturday, Jan. 29th, Dr. W. F. Harbaugh, Dentist, of Piqua, Ohio, murdered his wife, and then killed himself, leaving three interesting children.

This terrible tragedy is the result of a career of dissipation from the excessive use of intoxicating drink, in which he had indulged for two or three years. During this time it had been recognized by his friends, and by all who knew him, indeed, that he was rapidly going the downward road to ruin; though none had anticipated so sad an end. Previous to contracting this terrible habit Dr. H. was popular in the community in which he lived, and stood well in the profession, of which he had been for quite a number of years an honorable member.

He was a kind and affectionate husband and father when sober, but a very demon, it is said, when under the influence of liquor. A few short years ago none had a brighter prospect than Dr. H., and yet all was trampled in the dust at the behest of the accursed drink. The pleasure of those who indulge in the use of intoxicating drink is purchased at an awful cost, yet society seems determined to have it so.

